# ActiGraph GT3X+ and Actical Wrist and Hip Worn Accelerometers for Sleep and Wake Indices in Young Children Using an Automated Algorithm: Validation With Polysomnography

**DOI:** 10.3389/fpsyt.2019.00958

**Published:** 2020-01-14

**Authors:** Claire Smith, Barbara Galland, Rachael Taylor, Kim Meredith-Jones

**Affiliations:** ^1^ Department of Women’s and Children’s Health, University of Otago, Dunedin, New Zealand; ^2^ Department of Medicine, University of Otago, Dunedin, New Zealand

**Keywords:** actigraph, accelerometer, sleep, physical activity, 24-h, Polysomnography, children

## Abstract

**Objectives:** Our count-scaled algorithm automatically scores sleep across 24 hours to process sleep timing, quantity, and quality. The aim of this study was to validate the algorithm against overnight PSG in children to determine the best site placement for sleep.

**Methods:** 28 children (5–8 years) with no history of sleep disturbance wore two types of accelerometers (ActiGraph GT3X+ and Actical) at two sites (left hip, non-dominant wrist) for 24-h. Data were processed using the count-scaled algorithm. PSG data were collected using an in-home Type 2 device. PSG-actigraphy epoch sensitivity (sleep agreement) and specificity (wake agreement) were determined and sleep outcomes compared for timing (onset and offset), quantity [sleep period time (SPT) and total sleep time (TST)], and quality metrics [sleep efficiency and waking after sleep onset (WASO)].

**Results:** Overall, sensitivities were high (89.1% to 99.5%) and specificities low (21.1% to 45.7%). Sleep offset was accurately measured by actigraphy, regardless of brand or placement site. By contrast, sleep onset agreed with PSG using hip-positioned but not wrist-positioned devices (difference ActiGraph : PSG 21 min, P < .001; Actical : PSG 14 min, P < .001). The ActiGraph at the wrist accurately detected WASO and sleep efficiency, but under (−34 min, P < .001) and overestimated (5.8%, P < .001) these at the hip. The Actical under- and over-estimated these variables respectively at both sites. Results for TST varied ranging from significant differences to PSG of −26 to 21 min (ActiGraph wrist and hip respectively) and 9 min (ns) to 59 min for Actical (wrist and hip respectively).

**Conclusion:** Overall the count-scaled algorithm produced high sensitivity at the expense of low specificity in comparison with PSG. A best site placement for estimates of *all* sleep variables could not be determined, but overall the results suggested ActiGraph GT3X+ at the hip may be superior for sleep timing and quantity metrics, whereas the wrist may be superior for sleep quality metrics. Both devices placed at the hip performed well for sleep timing but not for sleep quality. Differences are likely linked to freedom of movement of the wrist *vs* the trunk (hip) during overnight sleep.

## Introduction

Short sleep duration, timing, poor quality, and high variability are characteristics of children’s sleep that are increasingly recognized as being associated with a wide range of adverse health outcomes including an increased risk of obesity (1). Accurate measurement of these sleep behaviors can be achieved using objective tools that estimate sleep and wake with sensors that measure body movement in terms of acceleration. These accelerometers are placed within small watch sized devices known as actigraphs (2). Accelerometer sensors are also used to estimate intensity of physical activity by processing movement into relevant components (sedentary, light, moderate, or vigorous physical activity) (3).

While sleep researchers traditionally refer to these devices as actigraphy devices, and physical activity researchers as accelerometers, they work on the same principle of using accelerometer sensors to detect motion. Devices used to measure physical activity are typically placed on the hip, whereas those used to measure sleep are usually placed on the wrist. These conventional practices stem from earlier validation studies when accelerometry sensors were uni-axial (sense movement in one direction only), so the sensors in physical activity devices were usually placed at the hip to be more sensitive to vertical acceleration associated with walking/running (4). Omnidirectional accelerometers are most sensitive in one plane, generally the vertical, but are also sensitive to movement in other directions, with the output being a composite of the signals. In contrast, triaxial accelerometers consist of three orthogonal accelerometer units that measure acceleration in each of the three planes separately, providing an output for each plane, as well as a composite measure (4).

The advantages of actigraphy/accelerometry are the relatively low cost and the unobtrusive nature of the device compared to laboratory or in-home PSG systems, making it an ideal tool for large-scale studies. However, most research to date has examined sleep and physical activity in isolation, requiring participants to either wear the device during the day to measure physical activity or just at night to measure sleep. Neither provide accurate estimation of sleep or physical activity given that participants do not put the device on as soon as they wake, and often take them off well before they go to bed (5). In order to improve compliance and wear time for both sleep and physical activity, protocols now recommend participants wear accelerometers over 24-h (6).

Estimating sleep from actigraphy requires computer algorithms to classify sleep and wake based on the assumption that the presence of movement indicates wakefulness and the absence of movement indicates sleep. Typically, algorithms vary by the population studied, device worn, and the site placement they were developed for (wrist or hip), but most work in a similar fashion: to define each minute of recorded activity as either a sleep or wake epoch by weighting the activity scores of the surrounding minutes. Various commercially available algorithms for assessing sleep are available (7), although the Sadeh algorithm (8) which has been validated in children and adolescents against the gold-standard for measuring sleep; polysomnography (PSG) (2, 8) is most commonly used in children. The major limitation of these algorithms is poor accuracy in detecting wake after sleep onset when subjects may be lying awake, but motionless, leading to overestimates of sleep (9). Conversely, when a person has very restless sleep, in the case of some sleep disorders, actigraphy can underestimate sleep (10). In addition, most sleep algorithms were developed for devices worn at the wrist and only a limited number of studies in adults (11–16) have demonstrated the accuracy of these algorithms compared to PSG when worn at the hip. To our knowledge, there are no studies that compare actigraphs worn at the hip to PSG in children.

We recently developed a count-scaled algorithm that automatically scores sleep and physical activity across a 24-h wear protocol (5). This count-scaled algorithm (17) uses a scaling process to standardize counts across the entire recording before epoch allocation, potentially giving the algorithm flexibility to apply to different accelerometers where count outputs differ due to different sensor sensitivities, or device placements.

To date we have validated the algorithm in infants during daytime napping and have shown accuracy rates of 85–86% against PSG (17). We have also reported agreements with parental diary data for overnight and 24-h sleep parameters in children from 6 months to 5 years (18). The current study extends this research for the measurement of nocturnal sleep in older children. The aims of this study are to: 1) validate the count-scaled algorithm against overnight PSG in children aged 5 to 8 years using two devices; the omnidirectional Actical and the triaxial ActiGraph GTX3+, 2) to compare different site placements (wrist *vs* hip) to determine the best placement site for measuring sleep, and 3) to compare the count-scaled automated algorithm to the most common algorithm used in child actigraphy, the Sadeh algorithm.

## Methods

### Subjects and Data Collection

Children were recruited from the general Dunedin (New Zealand) population by convenience sampling using a community newspaper, flyers on notice boards, and word of mouth. Children were aged 5 to 8 years at the time of recruitment. Ethical approval for the research was obtained from the University of Otago Human Ethics Committee (H15/025).

### Demographic and Anthropometric Data

Information was collected on participant’s age, sex, date of birth, and ethnicity using New Zealand census questions (19). The participants address was used to determine area based socio-economic status using the New Zealand Deprivation Index (NZDep Index, 2013) (20). At the first visit, height (cm) and weight (kg) were measured using standard techniques. Body mass index z-scores for age were calculated and cut-offs for overweight and obesity made according to WHO reference data (21).

### Sleep Disturbances Scale for Children (SDSC)

To assess children for sleep disturbance, parents completed the Sleep Disturbances Scale for Children (SDSC) (22). The SDSC is a 27-item validated inventory rated on a 5-point Likert-type scale that investigates the occurrence of sleep disorders over the last 6 months in children aged 6–16 years. The instrument’s purpose is to categorize sleep disorders in children. As well as giving an overall score the instrument uses six subdomains: disorders of initiating and maintaining sleep, sleep breathing disorders, disorders of arousal, sleep–wake transition disorders, disorders of excessive somnolence, and sleep hyperhidrosis. The sum of scores provides a total sleep score with a possible range from 26 to 130 (higher numerical values reflect a higher frequency of occurrence of symptoms). Higher scores indicate greater sleep difficulties, with total scores ≥56 signifying the presence of a clinically meaningful sleep disturbance.

### Actigraphs

**Figure 1:**
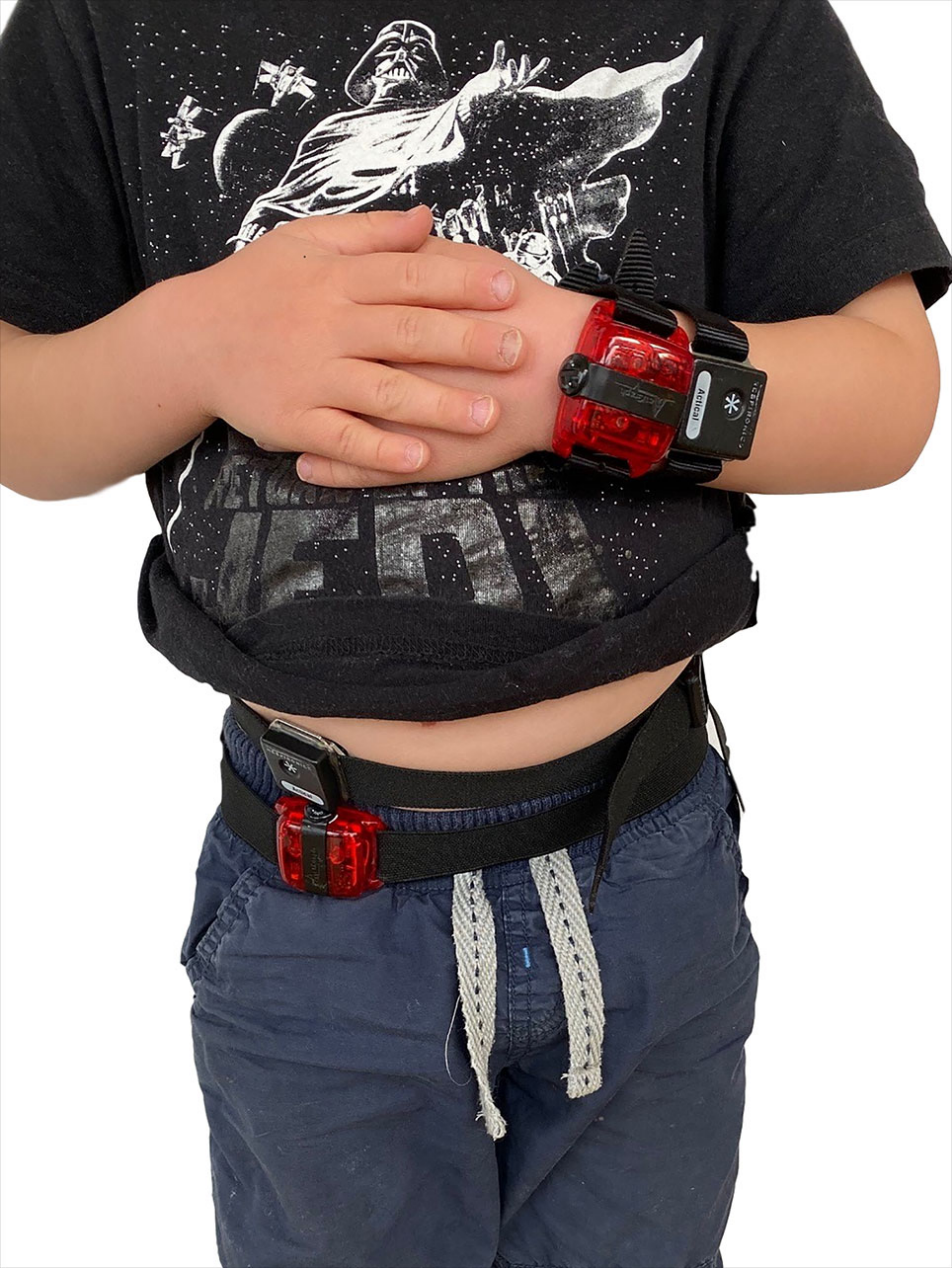
Site placements for wrist and waist worn ActiGraph GT3X+ and Actical accelerometers.

Children wore four accelerometers [two Actical (Philips Respironics Inc., Murrysville PA, USA) and two ActiGraph GT3X+ (ActiGraph, Pensacola, USA) accelerometers] on the right side of the hip and non-dominant wrist for approximately 36 h (**Figure 1**), including two overnight periods. All devices were initialized using 15 s epochs, and processed with the normal frequency filter (Actigraphs). The Actigraphs were initialized using Actilife (V 6.11.9), and the Acticals using Actical Version 3.0.The same computer was used to program the accelerometers and the PSG recording device and times were synchronized.

### Actigraphy Processing

Data were downloaded as.csv files and processed using the count-scaled algorithm developed in MATLAB (MathWorks, Natick, MA, USA) or the Sadeh algorithm (23) in ActiLife (ActiGraph, Pensacola, FL, USA). The count-scaled algorithm, described in greater detail elsewhere (5, 17) uses only the vertical axes outputs and scales each recording period for each participant relative to the mean value of all epochs that have non-zero counts. The algorithm is initiated using a “time flag” of 7:30 pm for sleep onset (night time sleep) and 6:00 am for sleep offset (morning wake). These flags were determined from the average bedtime and wake times that accurately reflected those for the age of our dataset, but can be modified for any age group or individual. To detect sleep and wake states, a weighted sum of the activity in the current minute, the preceding 4 min and the following 2 min is computed and then compared with the sleep–wake threshold of 1 (< 1 = sleep). The algorithm detects wake “events” as the last minute of 15 continuous minutes of sleep followed by 5 min of awake and sleep “events” as the start of 15 continuous minutes of sleep preceded by 5 min of awake. To detect the bedtime sleep “event” the algorithm first moves 3 h forward to detect the first sleep onset event. If sleep is not detected in the 3 h it moves 2 h backwards to identify the last sleep onset event. If a sleep event is not detected within the 3 h after or 2 h before the chosen bedtime, the chosen bedtime (e.g. 7:30 pm) is used. To detect sleep offset the algorithm performs in a similar way, but attempts to detect a wake time rather than a sleep time. All files in this study were processed using the automated mode, but the program does include an option for visual determination of sleep onset and offset as applied previously in other studies where sleep timing is not so predictable. The Sadeh algorithm is the most commonly used algorithm for sleep–wake scoring in children (24). The algorithm is: where SI is the sleep indicator of the current epoch (if SI ≥0, the current epoch is classified as sleep); μ is the mean activity on a 11‐min window centered on the current epoch; σ is the standard deviation of activity for the last 6 min; LogAct is the natural logarithm of the activity of the current epoch increased by 1 and nat is the number of epochs that satisfy the criterion 50 ≤ epoch activity <100 in an 11‐min window centered on the current epoch (23). As we used the Sadeh algorithm embedded within the ActiLife software which cannot be used to analyze Actical devices, we were only able to compare data from the Actigraphs.

### Home-Based Polysomnography

Overnight PSG was conducted on the second night of the accelerometer wear protocol and data were recorded using a Type 2 ambulatory sleep device (Embletta^®^ MPR with ST+ Proxy; Natus Medical, CA, USA) within participant’s homes at a sampling rate of 500 Hz and using guidelines of the American Academy of Sleep Medicine (25). The researcher began the PSG set up approximately 1 h before bedtime. The PSG included right and left electro-oculograms, four electroencephalograms (C4/M1, C3/M2, O2/M1, O1/M2), left and right submental electromyogram, thoracic and abdominal respiratory effort and ECG. Oxygen saturation was measured with pulse oximetry. Data were downloaded and analyzed using RemLogic software (Version 3.4, Embla Systems, Broomfield, CO, USA). Data were scored in 30-s epochs using AASM sleep staging criteria (Berry et al.). Sleep onset was considered the first epoch of sleep after lights out and sleep offset the last epoch of sleep. The study PSGs were scored visually by one author (CS) with a 94.3% inter-scorer reliability for sleep/wake on 12,317 against epochs scored by a second author (BG).

### Epoch-By-Epoch Comparison

The PSG and count-scaled actigraphy data were extracted epoch-by-epoch and aligned. To allow for comparison to the count-scaled algorithm the PSG 30 s epoch lengths were separated into two 15 s epochs. Depending on their agreement with PSG, each epoch was categorized as True Sleep, False Sleep, True Wake, or False Wake. Epoch concordance was calculated in terms of sensitivity (% sleep agreement), specificity (% wake agreement), and accuracy (% sleep and wake agreement). Because overnight recordings consist of many more sleep epochs than wake epochs (in a healthy subject, at least 85% of the epochs would correspond to “sleep” (26), to provide equal weights to this categorical data, a prevalence-adjusted bias-adjusted kappa (PABAK) was computed to counteract this (27). The interpretation of PABAK is the same as for kappa i.e. the Landis and Koch scale (28) is used to interpret the level of agreement where coefficients ≤0 indicate poor agreement; 0.01–0.20, slight agreement; 0.21–0.40, fair agreement; 0.41–0.60, moderate agreement; 0.61–0.80, substantial agreement; 0.81–1.00, almost perfect agreement. We did not analyze the Sadeh algorithm epoch-by as the algorithm uses 1-min epoch lengths compared the count-scaled algorithm which uses 15-s. We have however provided comparisons between the count-scaled algorithm and Sadeh algorithm for all summary sleep outcomes.

### Sleep Outcomes

**Figure 2:**
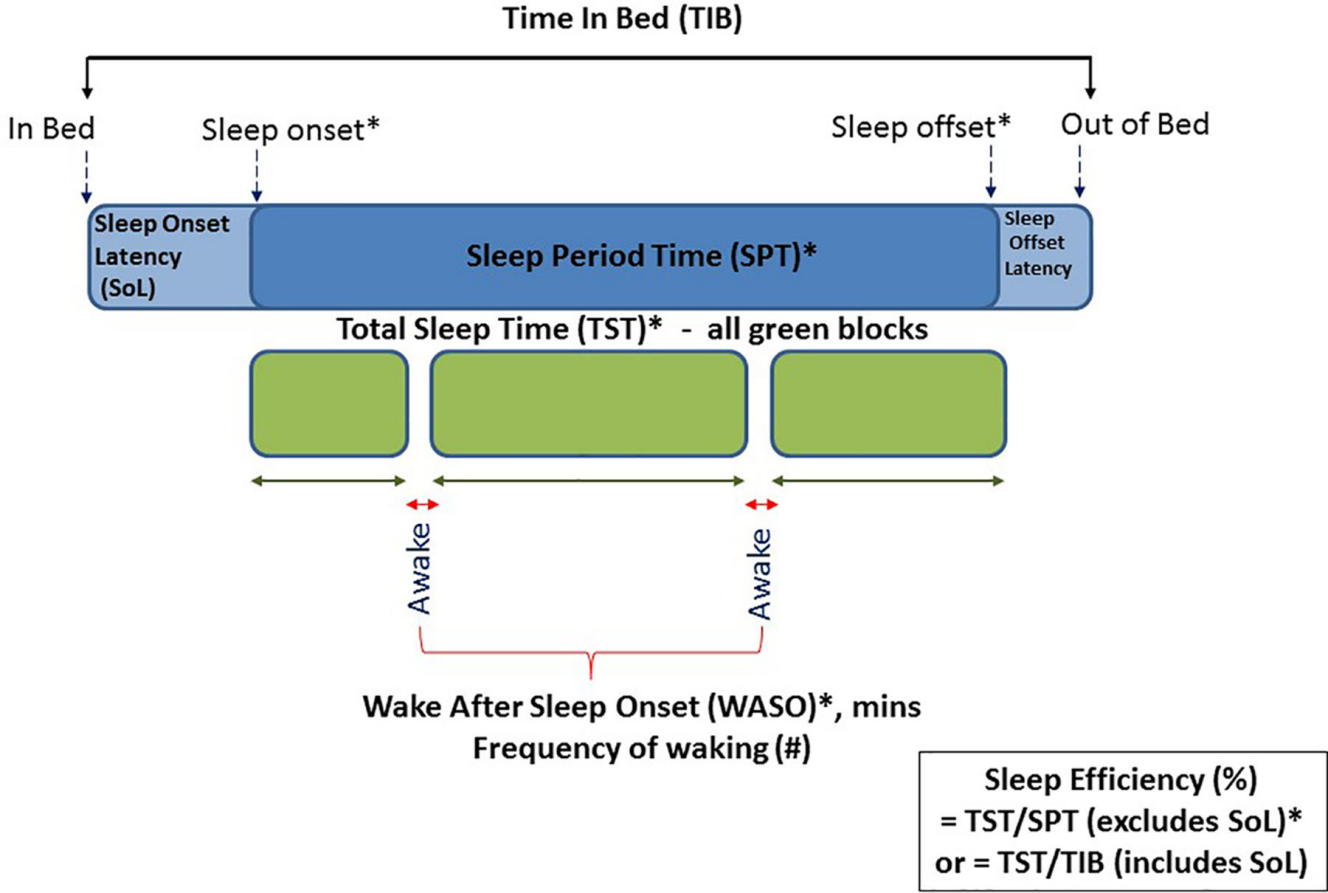
Schematic diagram of actigraphy and PSG data extracted across the night of sleep. Those marked with an asterisk were variables included in this validation study.

Sleep outcome variables were calculated from the PSG recordings and from the accelerometers located at both the wrist and hip, using the count-scaled algorithm and Sadeh algorithms as described above. The PSG and actigraphy data were analyzed separately by different researchers. Standard sleep variables were calculated for the period between sleep onset and sleep offset. Relevant sleep variables defined below included those related to dimensions of: a) sleep timing; sleep onset and sleep offset, b) sleep quantity; Sleep Period Time (SPT) and Total Sleep Time (TST), and c) sleep quality; sleep efficiency and waking after sleep onset (WASO). **Figure 2** gives a schematic of these variables extracted for both actigraphy and PSG.

- Sleep Onset: clock time of first consecutive minutes scored as evening sleep
- Sleep Offset: clock time of first consecutive minutes scored as morning wake
- Sleep Period Time (SPT): the elapsed time between sleep onset and sleep offset
- Wake after Sleep Onset (WASO): number of minutes scored as awake between sleep onset and offset
- Total Sleep Time (TST): represents true sleep time and is calculated as SPT minus WASO
- Sleep Efficiency: the percent of time asleep between sleep onset and offset and thus excludes sleep latency

Sleep outcomes were compared to PSG using paired t-tests for normally distributed data and Wilcoxon sign-rank test, for WASO, which was not normally distributed.

### Bland Altman

Bland Altman plots were used to examine the limits of agreement between sleep outcome variables of total sleep time and sleep efficiency measured by actigraphy devices against those measured by the gold-standard PSG. The Bland-Altman plot shows the difference between two measures on one axis against the average of two measures on the other axis (29). Upper (UALM) and lower limits (LALM) of agreement are calculated as the mean difference ± 1.96 × standard deviation. The limits of agreement are a measure of precision and show the range of values expected for 95% of individuals.

### Power Calculation

The sample size was powered to detect differences in our primary outcome variable which was mean total sleep time between PSG and accelerometry. Based on a mean (SD) for total sleep time by accelerometry of 660 (38) minutes in children aged 3–7 years (30), our study has 90% power at the 5% level of significance to detect a difference between methods of 30 min with 22 participants. This sample size also allows us to detect correlations between the two methods of at least r = 0.6.

## Results

### Study Participants

Twenty-eight participants with more than 5 h of PSG data were included in the epoch-by-epoch analyses and 23 participants with complete overnight PSG and congruent actigraphy were included in the comparison of sleep outcomes. **Table 1** provides a description of the participants. The mean age of participants was 7.2 years (SD 1.2), 17 (61%) were boys, the majority (n = 22, 79%) were of European ethnicity, and 14 (50%) resided in areas of low deprivation (NZDepIndex levels 1–3), 5 (18%) in areas of medium deprivation (levels 4–7), and 9 (32%) in areas of high deprivation (levels 8–10). Twenty five percent of participants were categorized as overweight and 4% as obese. The total SDSC score for all participants was within the normal (non-clinical) range with a mean (SD) score of 37.0 (5.7) and range, 26 to 52.

**Table1:** Demographic and health characteristics of participants (*n* = 28).

	n	%
Boys	17	61
Age (years)		
5	3	11
6	11	39
7	8	21
8	8	29
Ethnic group		
NZ Māori	2	7
European	22	79
Asian	4	14
Deprivation		
Low (1–3)	14	50
Med (4–6)	5	18
High (7–10)	9	32
BMI category		
Normal weight	20	71
Overweight	7	25
Obese	1	4
Sleep problem		
A small problem	1	4
Not a problem at all	27	96
Family position		
Middle	5	18
Oldest	12	43
Only	1	4
Youngest	10	36
< 15	7	25
15–30	10	36
30–45	5	18
45–60	5	18
> 60	1	4

BMI, body mass index.

### Sleep and Wake Epoch-By-Epoch Comparison

**Table 2** shows the sensitivity (epochs correctly identified as sleep), specificity (epochs correctly identified as wake) and accuracy (both sleep and wake epochs correctly identified) for the two accelerometers placed at the hip, and at the wrist. Mean values of overall accuracy were within a narrow range, from 86.0% (Actical at the wrist) to 90.2% (ActiGraph at the hip). Sensitivities achieved were also within a narrow range [95.7% (wrist positioned ActiGraph) to 99.5% (hip positioned Actical)]. Specificities however were more variable with the wrist positioned ActiGraph being highest 62.0%, with only 21.1% being achieved for the hip-positioned Actical. Epoch-by-epoch agreements using PABAK ranged from 0.74 (Actical hip) to 0.81 (ActiGraph wrist) with both devices showing moderate to substantial agreement overall and slightly higher agreement compared to PSG at the wrist than at the hip.

**Table 2:** Sensitivity, specificity, and accuracy of epoch-by-epoch comparisons with PSG of the two accelerometers placed at the hip and the wrist.

Device	Placement	n^a^	Accuracy	Sensitivity	Specificity	PABAK (95% CI)
			% (95% CI)	% (95% CI)	% (95% CI)	
ActiGraph GT3X+	Hip	28	88.2 (84.1, 91.3)	97.2 (96.1, 98.0)	41.6 (30.9, 53.2)	0.76 (0.75, 0.77)
ActiGraph GT3X+	Wrist	28	90.2 (86.3, 93.1)	95.7 (94.5, 96.4)	62.0 (47.0, 74.9)	0.81 (0.80, 0.81)
Actical	Hip	28	86.7 (82.7, 90.0)	99.5 (92.2, 99.6)	21.1 (15.3, 28.3)	0.74 (0.73, 0.74)
Actical	Wrist	27	86.0 (76.2, 92.2)	98.0 (97.3, 98.6)	45.7 (34.2, 57.3)	0.79 (0.65, 0.66)

a Participants with >5 h PSG and congruent actigraphy. PABAK, Prevalence-Adjusted Bias-Adjusted Kappa.

≤0 indicate poor agreement; 0.01–0.20, slight agreement; 0.21–0.40, fair agreement; 0.41–0.60, moderate agreement; 0.61–0.80, substantial agreement; 0.81–1.00, almost perfect agreement.

### Sleep Outcome Variables

#### Bivariate Correlations

Bivariate correlations for all sleep outcome variables according to PSG, actigraphy devices (ActiGraph GT3X+ and Actical) and placements (hip and wrist) are given in **Supplementary Tables S1**–**S5**. Sleep timing variables (onset and offset) were positively correlated (all significant excluding the wrist Actical), and sleep duration variables (TST and SPT) were significantly and positively correlated across all five matrices. Sleep quality variables of WASO and sleep efficiency were significantly negatively correlated. Other correlations were: sleep onset was significantly and negatively correlated with SPT and TST in all five matrices, whereas sleep offset correlated positively with SPT and TST i.e. the later the children went to sleep, the shorter their sleep was, and the later they woke up (sleep offset) the longer their sleep was in terms of both amount of sleep (TST) and opportunity for sleep (SPT). WASO significantly and negatively correlated with TST for the ActiGraph (both placements) and the Actical wrist i.e. the more waking between sleep onset and offset, the less total sleep. Although this trend was apparent within the PSG data, this was not significant. The hip placed Actical showed no relationship between TST and WASO.

#### ActiGraph GT3X+ *Vs* PSG

**Table 3** shows sleep and wake outcomes measured using the ActiGraph compared to PSG for the wrist and hip-positioned devices. Sleep onset recorded on the ActiGraph was significantly later (21 min) than PSG for the wrist positioned device, but not the hip, whereas sleep offset times were similar to PSG for both placements (hip 0:00 (0:26) min; wrist −0:05 (0:13) min). Consequently, the wrist placed device underestimated SPT (equating to the difference between onset and offset) by 27 min compared to PSG, whereas no significant differences were observed for the hip placement.

**Table 3:** Comparison of Actigraph GT3X measured sleep outcomes to PSG and comparison of wrist and hip positioned devices.

Sleep variable	Tool	Placement	n	Mean (SD)	Mean ∆ (95% CI) Act-PSG	*P* PSG-Act	*P* Hip *vs* wrist
Sleep onset (hh:min)	PSG	PSG	23	20:37 (0:38)			
	Actigraph GT3X	Hip	23	20:44 (0:43)	0:06 (−7, 20)	.328	
		Wrist	23	20:58 (0:33)	0:21 (13, 31)	**<.001**	**.022**
Sleep offset (hh:min)	PSG		23	6:50 (0:38)			
	Actigraph GT3X	Hip	23	6:49 (0:43)	0:00 (−11, 11)	.898	
		Wrist	23	6:45 (0:39)	−0:05 (−11, 1)	.086	.357
SPT^iii^ (min)	PSG		23	613 (43)			
	Actigraph GT3X	Hip	23	606 (40)	−7 (−22, 7)	.296	
		Wrist	23	586 (34)	−27 (−37, −16)	**<.001**	**.004**
WASO^iv^ median (IQR)	PSG		23	48 (19)			
(min)	Actigraph GT3X	Hip	23	12 (0, 41)	−34 (−14 to −9)	**<.001**	
		Wrist	23	48 (10, 78)	−7 (−36 to 19)	.784	**.001**
Sleep efficiency^v^ (%)	PSG		23	92.2 (91 to 93)			
Median (IQR)	Actigraph GT3X	Hip	23	98.0 (93 to 100)	5.8 (1.8 to 7.2)	**<.001**	
		Wrist	23	92.5 (86.5 to 98.1)	1.3 (−4.2 to 5.6)	.717	**.001**
Total sleep time^vi^ (min)	PSG		23	563 (46)			
	Actigraph GT3X	Hip	23	584 (64)	21 (1, 41)	**.043**	
		Wrist	23	538 (64)	−26 (−49, −3)	**.027**	**<.001**

^i^Comparison to PSG using paired t-tests (WASO compared using Wilcoxon rank sum test).

_ii_Comparison of hip and wrist measured sleep outcomes using paired t-tests (WASO compared using Wilcoxon rank sum test).

Wilcoxon Rank Sum tests used to compare actigraphy to PSG.

iii SPT is the time between sleep onset and offset.

iv WASO is the minutes of wake between sleep onset and sleep offset.

v Sleep efficiency = [(total sleep time−WASO)/sleep duration]×100.

vi Total sleep time is the time between sleep onset and offset with WASO removed. Bolded text indicates p < 0.05.

WASO measured using the ActiGraph at the wrist was not significantly different to PSG-derived WASO, whereas the hip positioned device underestimated WASO by 34 min. This impacted sleep efficiency such that hip placed devices overestimated efficiency by a median of 4.6%, whereas sleep efficiency derived from wrist worn devices was comparable to PSG. Since TST (the actual sleep time between onset and offset) uses all these metrics, the wrist device underestimated TST by 26 min, and the hip device overestimated TST by 21 min.

#### Hip *Vs* Wrist

The final column in **Table 3** shows P-values for the comparison of sleep outcomes for hip versus wrist placed devices. Sleep onset was significantly later measured by the wrist compared to the hip with no difference in sleep offset. There was no evidence of a difference for WASO or SPT between the wrist and the hip, however TST was significantly shorter for the hip. SE (%) was also significantly lower at the wrist (92.5%) compared to the hip (98%).

#### Count-Scaled *Vs* Sadeh

**Supplementary Tables S6** and **S7** show the comparison of the hip and wrist Actigraphs using the Sadeh algorithm compared to the count-scaled algorithm. At the hip, the only significant differences in performance (measured by difference to PSG) of the count-scaled algorithm compared to the Sadeh were for WASO (count-scaled underestimated WASO compared to the Sadeh; P = 0.029) and sleep efficiency (overestimated; P = 0.023), whereas all other metrics were comparable i.e. no significant differences encountered between the count-scaled and Sadeh performance for sleep onset, sleep offset, TST, or SPT. However at the wrist, the count-scaled algorithm performed better than the Sadeh on WASO and sleep efficiency (both differences P < .001) whereas all other metrics were comparable.

#### Actical *Vs* PSG

**Table 4** compares Actical-derived sleep measures compared to PSG for the wrist and hip positioned devices. Like the ActiGraph sleep onset using the Actical wrist positioned device was significantly later (14 min) than PSG, whereas the hip positioned device was comparable to PSG [−0:03 (0:13)]. Sleep offset time again was similar to PSG using both placements, as was SPT.

**Table 4:** Comparison of Actical measured sleep outcomes to PSG and comparison of wrist and hip positioned devices.

Sleep variable	Tool	Placement	n	Mean (SD)	Mean ∆ (SD) Act-PSG	*P* PSG-Act	*P* Hip *vs* wrist
Sleep onset (hh:min)	PSG		23	20:37 (0:38)			
	Actical	Hip	23	20:34 (0:53)	−0:03 (−18, 11)	.652	
		Wrist	22	20:54 (0:44)	0:14 (7, 22)	**<.001**	**.015**
Sleep offset (hh:min)	PSG		23	6:50 (0:38)			
	Actical	Hip	23	7:04 (0:50)	0:14 (−2, 31)	.084	
		Wrist	22	6:50 (0:51)	−0:01 (−16, 13)	.876	.119
SPT^iii^ (min)	PSG		23	613 (43)			
	Actical	Hip	23	631 (47)	18 (−4, 40)	.110	
		Wrist	22	597 (55)	−15 (−32, 2)	.075	**.005**
WASO^iv^ median (IQR)	PSG		23	48 (38 to 58)			
(min)	Actical	Hip	23	0 (0 to 7)	−45 (−58 to −21)	**<.001**	
		Wrist	22	17 (0 to 32)	−24 (−45 to −9)	**.003**	.121
Sleep efficiency^v^ median (IQR)	PSG		23	92.2 (90.0 to 92.7)			
(%)	Actical	Hip	23	99.9 (98.9 to 100)	7.6 (4.4 to 10.0)	**<.001**	
		Wrist	22	97.0 (90.4, 99.8)	4.3 (−1.9 to 7.0)	**.011**	.084
Total sleep time^vi^ (min)	PSG		23	563 (46)			
	Actical	Hip	23	622 (50)	59 (35, 83)	**<.001**	
		Wrist	22	571 (45)	9 (−14, 32)	.420	**.003**

^i^Comparison to PSG using paired t-tests (WASO compared using Wilcoxon rank sum test).

^ii^Comparison of hip and wrist measured sleep outcomes using paired t-tests (WASO compared using Wilcoxon rank sum test).

Wilcoxon Rank Sum tests used to compare actigraphy to PSG.

iii SPT is the time between sleep onset and offset.

iv WASO is the minutes of wake between sleep onset and sleep offset.

v Sleep efficiency = [(total sleep time−WASO)/sleep duration]×100.

vi Total sleep time is the time between sleep onset and offset with WASO removed. Bolded text indicates p < 0.05.

Both placements underestimated WASO (hip 45 min; wrist 24 min), resulting in an overestimation of sleep efficiency (hip 7.6%; wrist 4.3%). In calculating the SPT, these differences amounted to the hip positioned device overestimating SPT by 59 min, whereas the wrist device produced values comparable to PSG.

#### Hip *Vs* Wrist

The final column in **Table 4** shows P-values for the comparison of sleep outcomes for hip versus wrist placed Actical devices. The Actical device produced fewer sleep variable outcome differences between wrist and hip positions than the Actigraph GT3X+ (**Table 4**).

Sleep onset was significantly later measured at the wrist compared to the hip with no evidence of a difference for sleep offset. SPT and TST were shorter measured at the wrist. There was no evidence of a difference between the wrist and hip for WASO but the results showed a tendency for SE (%) to be lower at the wrist (97% *vs* 99%, P < 0.084).

#### Bland-Altman Plots

**Figure 3:**
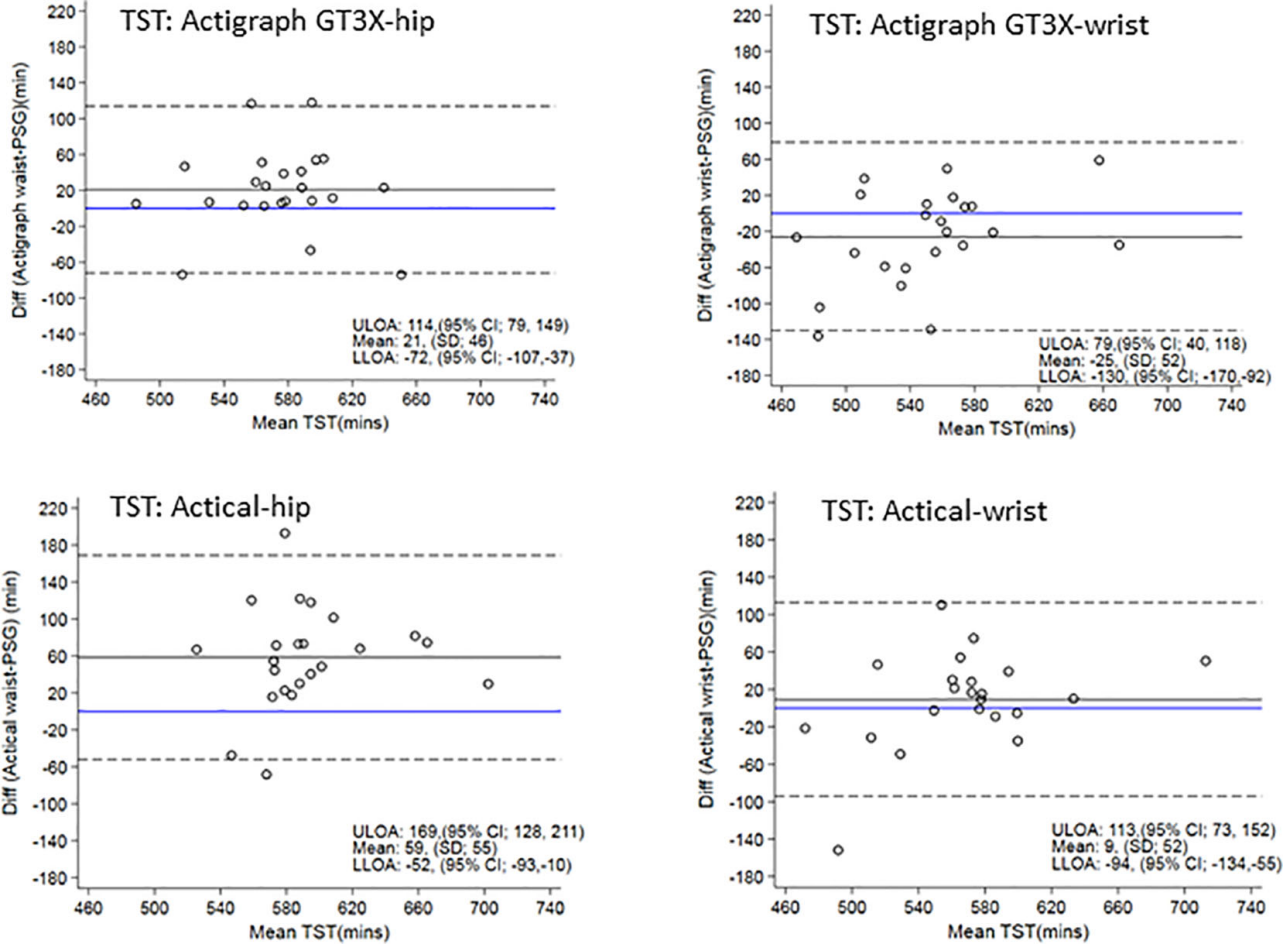
Bland Altman plots of TST differences between actigraphy and PSG are on the y-axis and means are on the x-axis. Perfect agreement is shown by the blue line crossing zero on the y-axis. Mean differences are represented by black solid lines and the upper and lower levels of agreement by dashed lines. ULOA, Upper Limit of Agreement; LLOA, Lower Limit of Agreement; TST, Total Sleep Time.

**Figure 3** displays Bland-Altman plots for the differences in TST for each device at the hip and wrist positions using the count-scaled algorithm against the gold standard PSG-derived measures. The plots illustrate a systematic positive bias (mean difference lines for each device were above zero) for TST estimated from the count-scaled algorithm for the ActiGraph and Actical positioned at the hip. The mean difference for the wrist positioned ActiGraph device was below zero indicating a systematic negative bias (underestimation) of TST measured using this device/placement. For the Actical at the wrist, the mean difference was closer to the line of identity (mean = 9 min) indicating less bias. The limits of agreement for all were large indicating a wide dispersal of differences with no trends apparent.

**Figure 4:**
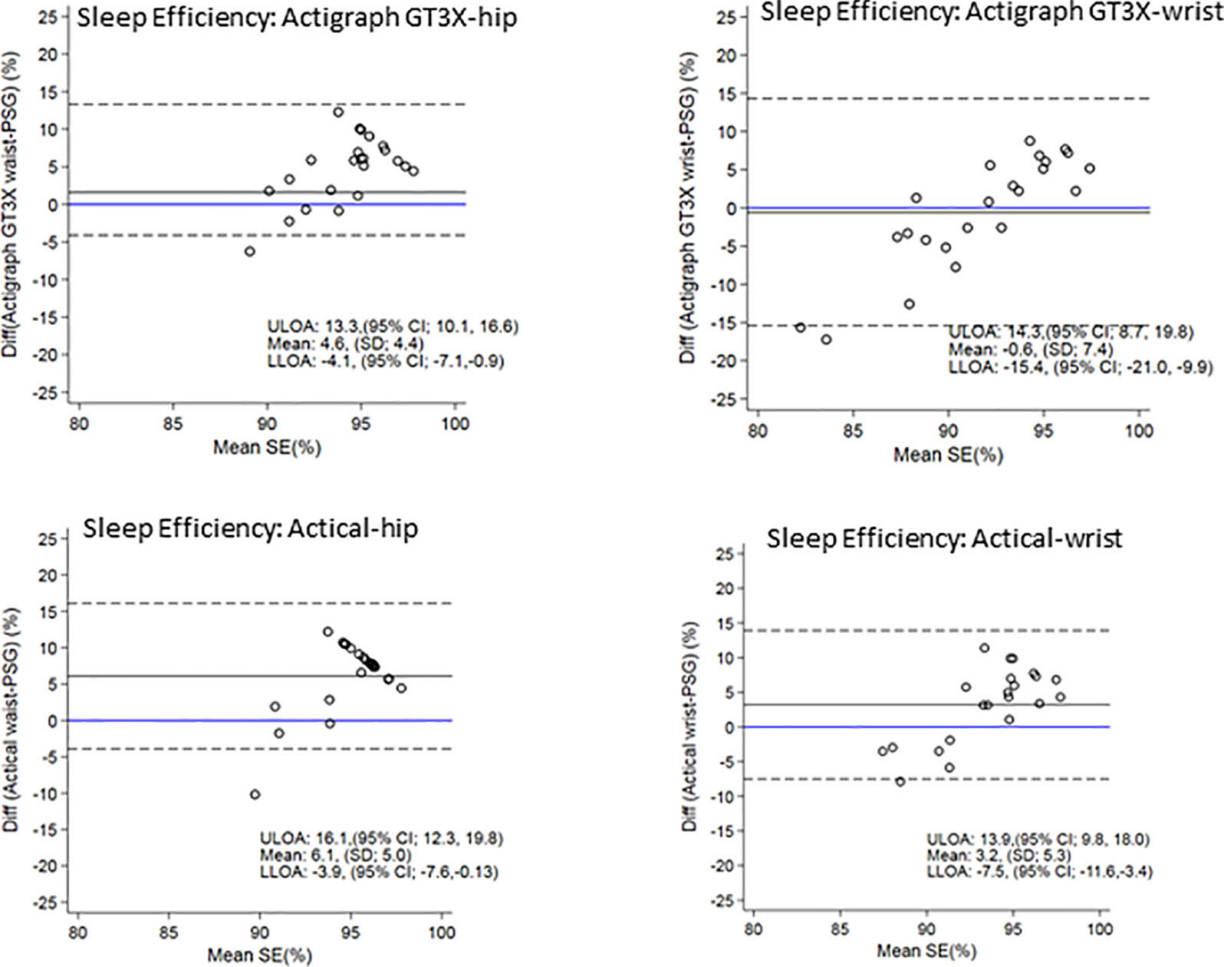
Bland Altman plots of TST differences between Acticals and PSG are on the y-axis and means are on the x-axis. Perfect agreement is shown by the blue line crossing zero on the y-axis. Mean differences are represented by black solid lines and the upper and lower levels of agreement by dashed lines. ULOA, Upper Limit of Agreement; LLOA, Lower Limit of Agreement; TST, Total Sleep Time.

**Figure 4** shows the Bland-Altman plots for sleep efficiency. The hip positioned ActiGraph and Actical positively overestimated sleep efficiency. There was less bias in sleep efficiency measured by the wrist ActiGraph (mean of −0.6% i.e. close to zero) and wrist (mean overestimation of 3%). All plots illustrate a trend for a larger positive difference as mean sleep efficiency increases i.e. the higher the sleep efficiency, the larger the discrepancies between PSG and actigraphy.

## Discussion

Our study demonstrates that overall, sleep variables estimated using the count-scaled algorithm and measured using both the ActiGraph and the Actical whether placed at the wrist or hip have high sensitivity for detecting sleep (96–99%) but poorer wake specificity (21–62%) in comparison with PSG. These findings are in agreement with a review of previous validation studies in children where one in five studies returned sensitivities above 80%, and more than half returned sensitivities below 60% (7). The level of accuracy must be considered within the context of the automated count-scaled algorithm, developed specifically for large-scaled studies. It does not rely on the concurrent collection of a sleep diary data which may have improved the sleep–wake agreement. However this has to be balanced against the time consuming nature of entering diary data for researchers handling large datasets.

Some differences were observed in relation to various sleep outcomes. Overall, sleep offset time estimates were very close to PSG (regardless of device or positioning) and compared well against the Sadeh algorithm, reflecting the preciseness of using our automated count-scaled algorithm to detect this metric in these actigraphy devices. This is perhaps not surprising given that young children are likely to produce bodily movements at both areas of device placement i.e. the trunk and limbs upon waking. The wrist placement for both devices produced later sleep onset than hip placement, the latter producing no differences in sleep onset time when compared against the gold standard PSG. These differences in sleep onset outcomes related to placement may reflect what happens when settling to sleep; a person’s trunk lies still, but the hands are still free to move, delaying the accuracy of the device at the wrist to detect the first identified period of sleep onset. Our actigraphy rules in processing required 15 min of sleep (epochs indicative of no or little movement) to be preceded by at least 5 min of awake (epochs indicative of movement) before marking sleep onset. The trunk can be assumed to be motionless during settling to sleep (if the person does not change position), and therefore a hip positioned device may detect sleep earlier than the wrist, and closer to the true sleep onset time.

The ActiGraph placed at the wrist was superior to the hip in terms of detecting waking after sleep onset and sleep efficiency in comparison with PSG. Taken together, and with the findings related to sleep timing above, our results suggest that researchers interested in sleep timing and sleep duration (sleep period time in this instance) would gain more accurate estimates using the hip placed ActiGraph at the expense of waking after sleep onset and sleep efficiency (sleep quality variables) that appear more reliably estimated at the wrist, and vice versa. It seems likely that the hands move more freely than the trunk during sleep and thus a wrist placement detects sleep disturbances more effectively. Hip placement may favor compositional data analysts who currently include all waking after sleep onset events as part of the sleep component of the 24-h day.

Overall, the Actical device placed at the hip measured sleep timing and duration (sleep period time) well. By contrast, metrics of sleep quality were relatively poor for this device at either placement site, underestimating WASO, and in keeping with the bivariate correlations, overestimating sleep efficiency (median 4.3% at wrist and 7.6% at the hip). Interestingly, at the wrist, this 4.3% overestimation of sleep efficiency is identical to previous estimates of sleep efficiency derived from a PSG validation study of 30 adolescents (mean age 17.6 years) using the exact same device and placement (31). Given that few studies have determined the accuracy of hip accelerometers to PSG for measuring sleep in children, this is an important finding and highlights the potential usefulness of the count-scaled algorithm for detecting sleep period time in large cohorts i.e. without the addition of sleep diaries.

The metric of total sleep time (TST) representing the true amount of time the child sleeps, reflects a combination of waking after sleep onset and the length of the sleep period (SPT) i.e. increased waking reduces TST but a longer sleep period time increases TST as reflected within the bivariate correlations. The device/placement that produced values closest to PSG-measured TST was the Actical placed at the wrist; however this was a function of both an underestimation of WASO and a shorter (albeit not significant) sleep period time. In the previous adolescent validation study of the wrist Actical (31), TST was underestimated by 31 min, although they did not provide data for either WASO or SPT to understand what was driving this. In the current study, all other device/placements produced TST values that were significantly different to PSG-derived values.

PSG validation studies of the ActiGraph published for both children and adults do not include WASO metrics for this device (16, 24). In part this may be due to algorithms within the proprietary software for analyzing ActiGraph sleep data. For children at least, awakenings are defined as one or more consecutive epochs (60 s) having count levels that indicate movement. Our experience is that this produces very high waking frequencies. Most actigraphy rules for children include 5 min or more of consecutive awakenings to define waking after sleep onset (32). The current program includes options to manually adjust sleep periods and sleep scoring rules. WASO is a critical metric in the sleep field with children’s night wakings being the most common sleep issue reported by parents (33). We suggest it is critical that software developers consider these shortcomings in future developments of proprietary software for 24-h accelerometry data.

All files in this dataset were analyzed using a count-scaled algorithm in automatic mode using time flags for the program to identify sleep onset and offset. This allows for hundreds of files to be analyzed at once, a major advantage for the processing of data from large-scale studies without the need for sleep diaries (5). In addition, the algorithm produces 24-h physical activity estimates that can be processed using user-defined cut-points for sedentary, and moderate to vigorous physical activity (34). Another automated sleep/wake algorithm has also been developed for a hip worn ActiGraph in children ActiGraph (35). The algorithm showed precise agreement with visual detection for sleep onset and offset, but has not yet been validated against gold-standard polysomnography. Despite referral in the paper to the algorithm detecting WASO (35), the data were not included, and therefore the precision of this variable against visual detection is unable to be ascertained.

This study cannot tell us which is the best placement to measure all activities across 24-h, as this requires validation of 24-h physical activity and sedentary behavior in concert with sleep. In moving forward, the ActiGraph is the one of the most widely used devices for measuring all behaviors of interest (36, 37). Our results do however suggest that for measuring sleep, different sleep dimensions will be impacted by placement site of the ActiGraph. Measuring variables such as sleep efficiency and waking after sleep onset are critical for measuring objective sleep quality, and therefore advances in this area for actigraphy are paramount given the move toward the importance of considering all sleep dimensions in regard to sleep health (38). Traditionally sleep quantity has been the key metric related to sleep health outcomes for children (1), probably related to its ease of measurement particularly in questionnaire or diary data, but there are now sleep quality recommendations for all age groups based on actigraphy and PSG data (39), and sleep timing and variability recommendations are a work in progress (40). All mean actigraphy values for sleep efficiency were in the recommended range for school-aged children i.e. ≥85% (39). For WASO, median durations measured by three of the four actigraph device–site combinations were in the appropriate (≤20 min) or uncertain recommendation ranges (21–40 min) for school-aged children; the Actical hip placement (WASO = 45 min) was just inside the inappropriate range recommendation i.e. 41 to 61+ min. Tracking of sleep architecture, including time spent in REM or NonREM sleep stages is not possible with accelerometry, as this requires the measurement of brain activity, eye movements, and muscle tension. Recent advances in consumer sleep trackers provide estimates of REM sleep, depth of sleep, and awake episodes across the night by including measures of heart rate alongside an accelerometer. The accuracy of these devices has not however been established.

Although debate remains regarding the best device placement for actigraphy, a review by Migueles et al. (41) suggests the hip site may produce more accurate estimates of physical activity (41), particularly with features from triaxial raw accelerometer signals that have narrowed the gap between physical activity energy expenditure estimates from wrist worn *vs* hip worn-devices (42, 43). A previous study in children using the ActiGraph devices for 24-h physical activity measures over 7 days found a higher compliance for wrist-worn versus hip-worn devices in 9–10 year-old children (44). Migueles et al. (41) also recommend different algorithms for estimating sleep-related behaviors for children and adolescents when devices are placed on the hip and the wrist; specifically the Tudor-Locke algorithm (45) for the hip and the Sadeh (46) for the wrist. For the hip, we suggest this would only apply for research concerned with when sleep onset and offset, as few sleep metrics are actually reported within the Tudor-Locke algorithm (45). Furthermore, this algorithm has not been validated against polysomnography.

The narrow age range of this study together with the exclusion of participants with sleep disturbance can be considered both a strength and a limitation. For validation purposes, this gave us a more homogenous sample to create greater precision in our estimates within the available sample size. Furthermore, keeping the age range narrow also reduces variability in sleep timing and duration, and excluding participants with sleep disturbance also reduces variability in other sleep quality metrics as well. However limitations are inherent in not knowing how well our algorithm performs across different age groups, or in those with significant sleep disturbance. In addition, the algorithm does not include sleep latency in the automated outputs, but if a sleep diary or event marker is used to mark time in bed, then sleep latency can be easily calculated. These points could be addressed in future research. Furthermore, it must also be noted that although relatively small mean differences were observed between the count-scaled algorithm and PSG for most sleep outcomes, when assessing TST, individual biases were still present as indicated by the wide limits of agreement (Actigraph hip/wrist range −130 to 114 min; Actical hip/wrist range −94 to 169 min).

In conclusion, the count-scaled algorithm (used in the fully automated mode) demonstrated good accuracy for detecting sleep–wake epochs and precision in estimating some sleep outcome variables in children using data from two actigraphy devices. While we could not achieve our aim to determine a single best site placement for precise estimates of *all* sleep variables, our findings suggest that, for the ActiGraph at least, the hip may be superior for sleep quantity metrics, whereas the wrist may be superior for sleep quality metrics. The Actical device was precise at detecting sleep timing regardless of placement, but at the expense of sleep quality metrics. Additional research is needed to validate sleep algorithms for wrist and the hip-worn accelerometers across all age groups, and reporting of all sleep metrics is paramount to be able to understand the intricacies and importance of device placement. Furthermore, research is needed to validate these and other devices for the assessment of 24-h movement behaviors, that is, sleep, sedentary behavior, and physical activity.

## Data Availability Statement

The datasets generated for this study are available on request to the corresponding author.

## Ethics Statement

The studies involving human participants were reviewed and approved by the University of Otago Human Ethics Committee (H15/025). Written informed consent to participate in this study was provided by the participants’ legal guardian/next of kin.

## Author Contributions

CS and KM-J are the Principle Investigators of the overall project. RT and KM-J conceived the idea for this study. CS and KM-J conducted the research. CS undertook all the data collection, PSG designed and undertook the statistical analyses. KM-J undertook all the accelerometry analyses. BG wrote the first and subsequent drafts of the manuscript and all authors critically revised the manuscript for important intellectual content. KM-J had primary responsibility for final content. All authors read and approved the final manuscript.

## Conflict of Interest

The authors declare that the research was conducted in the absence of any commercial or financial relationships that could be construed as a potential conflict of interest.

## Supplementary Material

The Supplementary Material for this article can be found online: https://www.frontiersin.org/articles/10.3389/fpsyt.2019.00958/full#supplementary-material
