# Supplementary material for: ActiGraph GT3X+ and Actical Wrist and Hip Worn Accelerometers for Sleep and Wake Indices in Young Children Using an Automated Algorithm: Validation With Polysomnography
Source: Front Psychiatry. 2020 Jan 14;10:958. doi: 10.3389/fpsyt.2019.00958 (PMC6970953; doi:10.3389/fpsyt.2019.00958)
Supplement: Supplementary file 1 [file DataSheet_1.docx]

**Supplementary Tables S1-S5**: Correlation matrices between sleep outcome variables

**Table S1.** Pairwise correlations for sleep outcomes measured by polysomnography

| Sleep Outcomes | Sleep Onset | Sleep Offset | SPT (mins) | WASO (mins) | Sleep Efficiency (%) | Total Sleep Time (min) |
| --- | --- | --- | --- | --- | --- | --- |
| Sleep Onset | 1.000 |  |  |  |  |  |
| Sleep Offset | 0.381* | 1.000 |  |  |  |  |
| Sleep Period Time (mins) | -0.556*** | 0.556*** | 1.000 |  |  |  |
| WASO (mins) | -0.011 | 0.003 | 0.012 | 1.000 |  |  |
| Sleep Efficiency (%) | -0.261 | 0.091 | 0.317 | -0.839*** | 1.000 |  |
| Total Sleep Time (min) | -0.560*** | 0.517** | 0.969*** | -0.209 | 0.542*** | 1.000 |
|  | | | | | | |
| **** p<0.01, ** p<0.05, * p<0.1* | | | | | | |

WASO, Wake After Sleep Onset; SPT, Sleep Period Time.

**Table S2.** Pairwise correlations for sleep outcomes measured by hip positioned Actigraph GT3X

| Sleep Outcomes | Sleep Onset | Sleep Offset | SPT (mins) | WASO (mins) | Sleep Efficiency (%) | Total Sleep Time (min) |
| --- | --- | --- | --- | --- | --- | --- |
| Sleep Onset | 1.000 |  |  |  |  |  |
| Sleep Offset | 0.491*** | 1.000 |  |  |  |  |
| Sleep Period Time (mins) | -0.463*** | 0.545*** | 1.000 |  |  |  |
| WASO (mins) | 0.143 | 0.194 | 0.059 | 1.000 |  |  |
| Sleep Efficiency (%) | -0.147 | -0.149 | -0.015 | -0.993*** | 1.000 |  |
| Total Sleep Time (min) | -0.490*** | 0.412** | 0.891*** | -0.400** | 0.447** | 1.000 |
|  | | | | | | |
| **** p<0.01, ** p<0.05, * p<0.1* | | | | | | |

WASO, Wake After Sleep Onset; SPT, Sleep Period Time.

**Table S3**. Pairwise correlations for sleep outcomes measured by wrist positioned Actigraph GT3X

| Sleep Outcomes | Sleep Onset | Sleep Offset | SPT (mins) | WASO (mins) | Sleep Efficiency (%) | Total Sleep Time (min) |
| --- | --- | --- | --- | --- | --- | --- |
| Sleep Onset | 1.000 |  |  |  |  |  |
| Sleep Offset | 0.478*** | 1.000 |  |  |  |  |
| Sleep Period Time (mins) | -0.334* | 0.669*** | 1.000 |  |  |  |
| WASO (mins) | 0.046 | -0.089 | -0.134 | 1.000 |  |  |
| Sleep Efficiency (%) | -0.108 | 0.167 | 0.270 | -0.987*** | 1.000 |  |
| Total Sleep Time (min) | -0.255 | 0.533*** | 0.788*** | -0.709*** | 0.794*** | 1.000 |
|  | | | | | | |
| **** p<0.01, ** p<0.05, * p<0.1* | | | | | | |

WASO, Wake After Sleep Onset; SPT, Sleep Period Time.

**Table S4.** Pairwise correlations for sleep outcomes measured by hip positioned Actical

| Sleep Outcomes | Sleep Onset | Sleep Offset | SPT (mins) | WASO (mins) | Sleep Efficiency (%) | Total Sleep Time (min) |
| --- | --- | --- | --- | --- | --- | --- |
| Sleep Onset | 1.000 |  |  |  |  |  |
| Sleep Offset | 0.560*** | 1.000 |  |  |  |  |
| Sleep Period Time (mins) | -0.602*** | 0.324* | 1.000 |  |  |  |
| WASO (mins) | -0.106 | 0.200 | 0.314* | 1.000 |  |  |
| Sleep Efficiency (%) | 0.070 | -0.252 | -0.318 | -0.989*** | 1.000 |  |
| Total Sleep Time (mins) | -0.517*** | 0.346* | 0.924*** | 0.147 | -0.145 | 1.000 |
|  | | | | | | |
| **** p<0.01, ** p<0.05, * p<0.1* | | | | | | |

WASO, Wake After Sleep Onset; SPT, Sleep Period Time.

**Table S5**. Pairwise correlations for sleep outcomes measured by wrist positioned Actical

| Sleep Outcomes | Sleep Onset | Sleep Offset | SPT (mins) | WASO (mins) | Sleep Efficiency (%) | Total Sleep Time (min) |
| --- | --- | --- | --- | --- | --- | --- |
| Sleep Onset | 1.000 |  |  |  |  |  |
| Sleep Offset | 0.252 | 1.000 |  |  |  |  |
| Sleep Period Time (mins) | -0.530*** | 0.687*** | 1.000 |  |  |  |
| WASO (mins) | 0.065 | -0.047 | -0.090 | 1.000 |  |  |
| Sleep Efficiency (%) | -0.165 | 0.189 | 0.288 | -0.987*** | 1.000 |  |
| Total Sleep Time (min) | -0.472** | 0.632*** | 0.908*** | -0.485** | 0.631*** | 1.000 |
|  | | | | | | |
| **** p<0.01, ** p<0.05, * p<0.1* | | | | | | |

WASO, Wake After Sleep Onset; SPT, Sleep Period Time.
