# Supplementary material for: ActiGraph GT3X+ and Actical Wrist and Hip Worn Accelerometers for Sleep and Wake Indices in Young Children Using an Automated Algorithm: Validation With Polysomnography
Source: Front Psychiatry. 2020 Jan 14;10:958. doi: 10.3389/fpsyt.2019.00958 (PMC6970953; doi:10.3389/fpsyt.2019.00958)
Supplement: Supplementary file 2 [file DataSheet_2.docx]

**Table S6:** Comparison of hip positioned Actigraph GT3X measured sleep outcomes to PSG using Sadeh algorithm and count scaled algorirthm

| Sleep Variable | Tool | n | Mean (SD) | Mean ∆ (95% CI)  Act-PSG | *P*^[[1]](#endnote-2)^  PSG-Act | | *P*  Count-scaled vs Sadeh^[[2]](#endnote-3)^ |
| --- | --- | --- | --- | --- | --- | --- | --- |
| Sleep Onset (hh:min) | PSG | 23 | 20:37 (0:38) |  |  | |  |
|  | Count scaled | 23 | 20:44 (0:43) | 6 (-7, 20) | .328 | | 0.242 |
|  | Sadeh | 23 | 20:54 (0:48) | 16 (8, 25) | **<0.001** | |  |
| Sleep Offset (hh:min) | PSG | 23 | 6:50 (0:38) |  |  |  |  |
|  | Count scaled | 23 | 6:49 (0:43) | -1 (-12, 10) | .898 | | 0.142 |
|  | Sadeh |  | 6:59 (0:8) | 9 (0,20) | .074 | |  |
| SPT^[[3]](#endnote-4)^ (mins) | PSG | 23 | 613 (43) |  |  |  |  |
|  | Count scaled | 23 | 606 (40) | -7 (-22, 7) | .296 | | 0.976 |
|  | Sadeh | 23 | 606 (39) | -7 (-20, 6) | .297 | |  |
| WASO^[[4]](#endnote-5)^ Median (IQR) | PSG | 23 | 48 (19) |  |  | |  |
| (mins) | Count scaled | 23 | 12 (0 to 41) | -34 (-45 to -9) | **<.001** | | **0.023** |
|  | Sadeh |  | 37 (13 to 82) | -2 (33 to 13) | 0.543 | |  |
| Sleep Efficiency^[[5]](#endnote-6)^ (%) | PSG | 23 | 92.2 (91 to 93) |  |  | |  |
| Median (IQR) | Count scaled | 23 | 98.0 (93 to 100) | 5.8 (1.8 to 7.2) | **<.001** | | **0.029** |
|  | Sadeh |  | 93 (89 to 99) | 0.5 (-2 to 6) | 0.465 | |  |
| Total Sleep Time^[[6]](#endnote-7)^ (mins) | PSG | 23 | 563 (46) |  |  |  |  |
|  | Count scaled | 23 | 584 (64) | 21 (1, 41) | **.043** | | 0.052 |
|  | Sadeh |  | 562 (46) | -2 (-22, 19) | 0.866 | |  |

**Table S7:** Comparison of wrist positioned Actigraph GT3X measured sleep outcomes to PSG using Sadeh algorithm and count scaled algorirthm

| Sleep Variable | Tool | n | Mean (SD) | Mean ∆ (95% CI)  Act-PSG | *P*^[[7]](#endnote-8)^  PSG-Act | | *P*  Count-scaled vs Sadeh^[[8]](#endnote-9)^ |
| --- | --- | --- | --- | --- | --- | --- | --- |
| Sleep Onset (hh:min) | PSG | 23 | 20:37 (0:38) |  |  | |  |
|  | Count scaled | 23 | 20:58 (0:33) | 21 (13, 31) | <0.001 | | 0.777 |
|  | Sadeh | 23 | 21:00 (0:42) | 23 (12, 32) | <0.001 | |  |
| Sleep Offset (hh:min) | PSG | 23 | 6:50 (0:38) |  |  |  |  |
|  | Count scaled | 23 | 6:45 (0:39) | -5 (-11, -1) | 0.086 | | 0.142 |
|  | Sadeh | 23 | 7:00 (0:38) | 9 (-1, 20) | 0.069 | |  |
| SPT^[[9]](#endnote-10)^ (mins) | PSG | 23 | 613 (43) |  |  |  |  |
|  | Count scaled | 23 | 586 (34) | -27 (-37, -16) | <0.001 | | 0.043 |
|  | Sadeh | 23 | 600 (32) | -13 (-30, 4) | 0.131 | |  |
| WASO^[[10]](#endnote-11)^ Median (IQR) | PSG | 23 | 48 (34 to 59) |  |  | |  |
| (mins) | Count scaled | 23 | 48 (10 to 78) | -7 (-36 to 20) | 0.784 | | <0.001 |
|  | Sadeh | 23 | 88 (56 to 128) | 44 (16 to 80) | <0.001 | |  |
| Sleep Efficiency^[[11]](#endnote-12)^ (%) | PSG | 23 | 92.2 (91 to 93) |  |  | |  |
| Median (IQR) | Count scaled | 23 | 92.5 (86.5 to 98.1) | 1.3 (-4.2 to 5.6) | 0.927 | | 0.001 |
|  | Sadeh | 23 | 85.0 (80 to 88) | -7 (-13 to -2) | <0.001 | |  |
| Total Sleep Time^[[12]](#endnote-13)^ (mins) | PSG | 23 | 563 (46) |  |  |  |  |
|  | Count scaled | 23 | 538 (64) | -26 (-49, -3) | **0.027** | | 0.052 |
|  | Sadeh | 23 | 507 (50) | -56 (-80, -32) | **<0.001** | |  |

1. Comparison to PSG using paired ttests (WASO compared using Wilcoxon rank sum test) [↑](#endnote-ref-2)
2. Comparison of the mean differences of Count-scale and Sadeh algorithms using paired ttests (WASO compared using Wilcoxon rank sum test) [↑](#endnote-ref-3)
3. Wilcoxin Rank Sum tests used to compare actigraphy to PSG

   ^iii^ SPT is the time between sleep onset and offset

   ^iv^ WASO is the minutes of wake between sleep onset and sleep offset

   ^v^ Sleep efficiency = ((total sleep time-WASO)/sleep duration))*100

   ^vi^ Total Sleep Time is the time between sleep onset and offset with WASO removed [↑](#endnote-ref-4)
4. [↑](#endnote-ref-5)
5. [↑](#endnote-ref-6)
6. [↑](#endnote-ref-7)
7. Comparison to PSG using paired ttests (WASO compared using Wilcoxon rank sum test) [↑](#endnote-ref-8)
8. Comparison of the mean differences of Count-scale and Sadeh algorithms using paired ttests (WASO compared using Wilcoxon rank sum test) [↑](#endnote-ref-9)
9. Wilcoxin Rank Sum tests used to compare actigraphy to PSG

   ^iii^ SPT is the time between sleep onset and offset

   ^iv^ WASO is the minutes of wake between sleep onset and sleep offset

   ^v^ Sleep efficiency = ((total sleep time-WASO)/sleep duration))*100

   ^vi^ Total Sleep Time is the time between sleep onset and offset with WASO removed [↑](#endnote-ref-10)
10. [↑](#endnote-ref-11)
11. [↑](#endnote-ref-12)
12. [↑](#endnote-ref-13)
